# Serum Lectin-Like Oxidized-Low Density Lipoprotein Receptor-1 and Adiponectin Levels Are Associated With Coronary Artery Disease Accompanied With Metabolic Syndrome

**DOI:** 10.5812/ircmj.12106

**Published:** 2014-08-05

**Authors:** Ali Sheikh Md Sayed, Zhenyu Zhao, Lanyan Guo, Fei Li, Xu Deng, Hai Deng, Ke Xia, Tianlun Yang

**Affiliations:** 1 Department of Cardiology, Xiangya Hospital, Central South University, Changsha, China; 2 Institute of Clinical Pharmacology, Central South University, Changsha, China; 3 Xijing Hospital, Fourth Military Medical University, Xi An, China; 4 Center for Vascular Biology and Inflammation, Cardiovascular Division, Department of Medicine, Brigham and Women’s Hospital, Harvard Medical, Boston, USA; 5 Institute of Hypertension, Central South University, Changsha, China

**Keywords:** LOX-1, Coronary Artery Disease, Adiponectin, Metabolic Syndrome

## Abstract

**Background:**

Coronary artery disease (CAD) is a major public health problem for developed and developing countries and is the single leading cause of death worldwide.

**Objectives:**

There is very few evidence regarding changes of both serum Lectin-like oxidized-low density lipoprotein receptor-1 (LOX-1) and adiponectin in patients with CAD accompanied with metabolic syndrome (MS). Here we aimed to evaluate serum levels of LOX-1 and adiponectin in patients with CAD accompanied with MS.

**Patients and Methods:**

Thirty patients with coronary artery disease without metabolic syndrome, 30 patients with coronary artery disease and metabolic syndrome, 30 ones with metabolic syndrome and 30 healthy subjects were enrolled. For all subjects, a questionnaire was filled to collect data, and peripheral blood samples were collected aseptically from the antecubital vein to measure serum Lectin-like oxidized-low density lipoprotein receptor-1 and adiponectin levels by enzyme-linked immunosorbent assay.

**Results:**

Serum LOX-1 level was highest in CAD + MS group; the difference between control and disease groups was statistically significant (P < 0.001). Adiponectin level had the lowest value in CAD + MS group; the difference between control and disease groups was statistically significant (P < 0.05). No significant differences were observed in serum Lectin-like oxidized-low density lipoprotein receptor-1and adiponectin in patients with different ages and gender. Serum LOX-1 level was changed negatively and linearly (R2 = 0.721) correlated with adiponectin level in different groups.

**Conclusions:**

Patient with CAD and MS had higher risk than those with only CAD because of lipid and glucose metabolism abnormalities. Combination measurements of serum LOX-1 and adiponectin levels may be helpful to evaluate the severity of CAD together with MS.

## 1. Background

Proinflammatory oxidized low-density lipoprotein (ox-LDL) is the unifying link between lipid accumulation and inflammation in the vessel wall and ox-LDL in plasma and within atherosclerotic lesions is strongly associated with CAD and vulnerable plaques (1). Endothelial dysfunction elicited by ox-LDL and its receptor has been shown to play a crucial role in the pathogenesis of atherosclerosis. LOX-1, a novel ox-LDL receptor with 50 kDa type II transmembrane glycoprotein comprising 273 amino acids, was cloned on bovine aortic endothelial cells in 1997 (2). LOX-1 supports binding, internalization and proteolytic degradation of ox-LDL when cells accepted stimulus from proinflammatory cytokine. Increased LOX-1 mRNA and protein expression in endothelial cells, macrophages, vascular smooth muscle cells and platelets are stimulated by angiotensin II and proinflammatory cytokine such as tumor necrosis factor alpha, which causes apoptosis, decreases nitric oxide synthase expression and stimulates reactive oxygen species (3). Several reports revealed that elevated serum levels of ox-LDL is associated with CAD (4). Soluble form of LOX-1 can be found on the cell surface into culture media. It has been reported that serum LOX-1 level is specifically elevated in acute coronary syndromes. Peak values of LOX-1 are observed earlier than those of Troponin T in acute coronary syndromes (5). Recently, many authors have shown that LOX-1 can be used as a good new biomarker in the diagnosis and prognosis of patients with coronary artery disease (6). Ox-LDL is also associated with MS and total ox-LDL levels were higher in subjects with MS than those without it (7). Circulating amount of oxidation-related moiety on low density lipoprotein has been shown to be effective to predict or diagnose MS and cardiovascular diseases (8). Combination of serum adiponectin level and MS is closely associated with CAD (9). Nevertheless, rare studies revealed the effect of LOX-1 in predicting MS.

Adipose tissue-derived factors such as free fatty acids, several cytokines including tumor necrosis factor alpha, interleukins, leptin, and adiponectin are the characters of MS. Increasing attention has been paid to the direct vascular effects of plasma proteins that originate from adipose tissue, especially adiponectin. Adiponectin is the most abundant adipokine secreted by adipose cells, which may couple regulation of insulin sensitivity with energy metabolism. Adiponectin is a 30-kDa protein containing an N-terminal collagenous domain and a C-terminal globular domain. Decreased plasma adiponectin levels is observed in patients with diabetes, MS, and CAD (10). Although the mechanisms underlying antiinflammatory properties of adiponectin are not well understood, adiponectin antiinflammatory and antiatherogenic properties may be related, in part, to its ability to stimulate production of nitric oxide from vascular endothelium (11). Low plasma adiponectin level is significantly correlated with endothelial dysfunction and could be a useful marker for early-stage atherosclerosis. Very recently, it has been demonstrated that a low adiponectin level combined with an advanced age, male gender, diabetes mellitus (DM) and hypertension (HT) is directly associated with multivessel coronary atherosclerosis disease (12). Plasma concentrations of adiponectin are significantly lower in patients with acute coronary syndromes than those with stable angina and the control group (13). In addition, adiponectin is associated with a decreased risk of coronary heart disease events in the same cohort men with diabetes (14). Low adiponectin levels are also associated with MS (15). 

MS is a complex of interrelated risk factors for cardiovascular disease (16). At a clinical level, patients with MS need to be identified to reduce their multiple risk factors. Nonetheless, patients with MS are at twice risk of developing cardiovascular disease over the next 5 to 10 years as individuals without the syndrome. MS diagnosis according to the World Health Organization criteria could be made based on several markers of insulin resistance together with some additional risk factors, including obesity, hypertension, high triglyceride level, reduced high-density lipoprotein cholesterol level, or microalbuminuria. However, combined serum markers such as LOX-1 and adiponectin in CAD accompanied with MS are still unknown.

## 2. Objectives

Our study aimed to examine serum LOX-1and adiponectin levels in patients with CAD and MS.

## 3. Patients and Methods

### 3.1. Study Approval

In this clinical study, all participants signed a written consent prior to their enrollment, which was undertaken with the approval of the local ethics committee of the Cardiology Department of Xiangya Hospital (Xiangya Medical College, Central South University, code-10533). Following ethical approval, permission obtained to perform the study and access to the patients and their records.

### 3.2. Study Population

Patients admitted to Cardiology Department of Xiangya Hospital, Central South University, China, were enrolled in this study from 8 August 2009 to 30 June 2010. Thirty patients with CAD with a mean age of 66 years (23 males and 7 females), 30 patients with CAD and MS with a mean age of 61 years (13 males and 17 females), and 30 patients with MS with a mean age of 53 years (23 males, 7 females) were enrolled as disease groups. For control group, 30 healthy subjects with a mean age of 51 years (22 males, 8 females) were enrolled from the Xiangya Medical Center. Both the patients and controls were selected from the Han ethnic population born and currently living in Changsha City of Hunan Province, People’s Republic of China. The main purpose of this study was to compare the LOX-1 and Adiponectin levels between patients with CAD + MS and those with only CAD; here we used the two-sample mean comparison formula to calculate the sample size. From the literatures, the level of Adiponectin in patients with CAD and MS was about 7.0 μg/mL, and the level of Adiponectin in patients with CAD without MS was 11.0 μg/mL, and the standard deviation (σ) was almost 4.0 μg/mL, the difference between the two populations (δ) was 4.0 μg/mL. Let α = 0.05 and β = 0.10 and the number calculated as 23.

$$n_{1}=n_{2}{=2[\frac{(u_{\frac{\alpha }{2}})}{\frac{\delta }{\sigma }}]}^{2}+\frac{1}{4}{u_{\alpha ⁄2}}^{2}=2\times [{\frac{\left(1.96+1.282\right)}{\frac{4.0}{4.0}}]}^{2}+\frac{1}{4}\times 1.96^{2}\approx 23$$

From the inspection results of patients in this study, the level of LOX-1 in patients with CAD and MS was about 200 pg/mL, and the level of LOX-1 in patients with CAD without MS was 400 pg/mL, and the standard deviation (σ) was almost 100 pg/mL, let the difference between two populations (δ) was 200 pg/mL and the number calculated as 12.

$$n_{1}=n_{2}{=2[\frac{(u_{\frac{\alpha }{2}}+u_{\beta })}{\frac{\delta }{\sigma }}]}^{2}+\frac{1}{4}{u_{\alpha ⁄2}}^{2}=2\times [{\frac{\left(1.96+1.282\right)}{\frac{200.0}{100.0}}]}^{2}+\frac{1}{4}\times 1.96^{2}\approx 12$$

In the actual operation, sample size in each group was increased to 30 to reduce the sample bias. Inclusion criteria for control group were; age of 30-75 yours, physical and clinical examination report with normal findings; no history of hypertension or not taking any antihypertensive medications and diabetes, and no acute or chronic disease condition; all blood and biochemical reports such as complete blood count, lipid profile, liver function, and kidney function had normal results; echocardiography and ultrasonography reports had normal findings. Selection of patients with CAD was based on clinical diagnosis. Based on coronary angiography findings regarding left anterior descending artery, left circumflex artery and right coronary artery, patients were considered to have CAD if coronary artery stenosis (at least one of them) was equal to or more than 50%. According to the American Heart Association, coronary artery stenosis was divided into less than or equal to 25%, 50%, 75%, 90%, 99%, 100% (occlusion). Symptoms of CAD were recorded and classified according to the Canadian Cardiovascular Society and New York Heart Association. Patients with acute coronary syndrome or acute myocardial infarction or CAD patients with acute or chronic heart failure were excluded in this group. Selection of patients with MS was based on clinical diagnosis. According to the International Diabetes Federation and the National Heart, Lung, and Blood Institute, there are five risk factors for MS as dysglycemia, raised blood pressure, elevated triglyceride levels, low levels of high-density lipoprotein cholesterol and obesity (particularly central adiposity). Patients were diagnosed as MS if three of the above five mentioned risk factors were present. Patients with CAD were excluded in this group. 

### 3.3. Data Collection

For all the study subjects, an standardized questionnaire was filled to collect data and comprehensive physical and medical examinations were performed after 12 hours fasting in the morning, such as height (m), weight (kg), blood pressure (mmHg) and body mass index (BMI). 

### 3.4. Biochemical Measurements

Peripheral blood sample was aseptically collected from the antecubital vein for biochemical measurements. Fasting blood glucose, total cholesterol, triglycerides, low-density lipoprotein cholesterol and high-density lipoprotein cholesterol, liver function, kidney function and high-sensitivity C-reactive protein (hs-CRP) were evaluated using an automatic analyzer (Hitach75, Tokyo, Japan).

### 3.5. Measurements of Serum LOX-1 and Adiponectin

Another four mL of blood was also taken and placed in ethylenediaminetetraacetic acid tubes, centrifuged at 1000 rpm for 5 minutes, then the serum was collected and stored at -80℃ for further measurements. The concentrations of LOX-1 and adiponectin were determined by commercial ELISA kit (Shanghai Yueyan Biology Technology Co, China) and the color changes were measured spectrophotometrically at a wavelength of 450 nm by comparing the O.D. of the samples to the standard curve. Here we used purified Human LOX-1 and adiponectin antibody respectively.

### 3.6. Statistical Analysis

Data was analyzed and graphs were constructed by statistical program, SPSS-16.0 and Microsoft Excel. T-test, One-way ANOVA and Student-Newman-Keuls post hoc test, chi-square test and Bonferroni post hoc test, Kruskal-Wallis H test and Student-Newman-Keuls post hoc test, Pearson’s correlation and multiple linear regressions were used. Values in figure and table were shown as mean ± SD and proportion. P < 0.05 was considered as statistically significant.

## 4. Results

### 4.1. General Clinical Information and Biochemical Indicators

General clinical information and biochemical values were shown in Table 1. Systolic and diastolic blood pressure in CAD + MS group (142 ± 9 mmHg/82 ± 7 mmHg), MS group (136 ± 6 mmHg/74 ± 7 mmHg) and CAD group (129 ± 10 mmHg/77 ± 7 mmHg) were higher than control group (124 ± 8 mmHg/72 ± 5 mmHg), respectively; the differences were statistically significant (P < 0.05). Body mass index in CAD + MS group (27.2 ± 1 kg/m^2^), MS group (25.6 ± 1 kg/m^2^) and CAD group (23.7 ± 1.3 kg/m^2^) were higher than control group (21.1 ± 1.5 kg/m^2^), which were significantly different (P < 0.05). Serum triglyceride (2.1 ± 1.4 mmol/L), fasting blood sugar (6.2 ± 0 mmol/L), Aspartate amino-transferase (23.5 ± 4.8 U/L), Alanine amino-transferase (21.2 ± 4 U/L), creatinine (89.3 ± 16 mg/L), uric acid (314.7 ± 55.1 mmol/L) and hs-CRP (12.0 ± 8.5 mg/L) levels were higher in CAD + MS group compared with control group. Triglyceride (0.84 ± 0.3 mmol/L), fasting blood sugar (4.5 ± 0.3 mmol/L), aspartate amino-transferase (11.2 ± 3.6 U/L), alanine amino-transferase (14.5 ± 4.5 U/L), creatinine (71.0 ± 9.8 mg/L), uric acid (251.9 ± 42 mmol/L) and hs-CRP (0.7 ± 0.8mg/L), were significantly different (P < 0.05). The CAD group had higher blood urea nitrogen (5.5 ± 0.9 mmol/L) level than the CAD + MS (5.3 ± 1 mmol/L) group, MS group (4.8 ± 1.4 mmol/L) and the control group (3.9 ± 0.6 mmol/L), which were significantly different (P < 0.05).

### 4.2. Role of Gender Variation in LOX-1 and Adiponectin Level in Different Groups

Serum LOX-1 and adiponectin levels of male and female participants in different groups were shown in Table 2. Serum LOX-1 levels in different groups were as follows; control group: male (86.6 ± 8.2 pg/mL), female (88.7 ± 9.9 pg/mL); CAD group: male (195.0 ± 41.5 pg/mL), female (200.0 ± 39.4 pg/mL); MS group: male (137.2 ± 11.5 pg/mL), female (149.7 ± 18.8 pg/mL) and CAD + MS groups: male (464.4 ± 100.8 pg/mL), female (414.3 ± 107.5 pg/mL). Adiponectin levels in different groups were as follows; control group: male (515.1 ± 30.0 µg/L), female (499.1 ± 25.4 µg/L); CAD group: male (370.5 ± 20.9 µg/L), female (368.0 ± 21.4 µg/L); MS group: male (456.9 ± 23.8 µg/L), female (436.7 ± 23.1 µg/L) and CAD + MS group: male (264.8 ± 20 µg/L), female (262.8 ± 31.4 µg/L). No significant differences were observed in plasma LOX-1 and adiponectin levels between male and female participants in each group (P > 0.05).

### 4.3. Role of Age Variation in LOX-1 and Adiponectin Levels in Different Groups

LOX-1 and adiponectin levels of CAD and MS patients in different age groups were shown in Table 3. Serum LOX-1 levels in different groups were as follows: control group: ≤ 50 years (85.5 ± 5.5 pg/mL), ≤ 70 years (87.8 ± 11.5 pg/mL), > 70 years (92.5 ± 6.8 pg/mL); CAD group: ≤ 50 years (232.4 ± 8.1 pg/mL), ≤ 70 years (178.6 ± 28.6 pg/mL), > 70 years (220.5 ± 46.3 pg/mL ); MS group: ≤ 50 years (139.4 ± 11.1 pg/mL), ≤ 70 years (142.9 ± 18.3 pg/mL), > 70 years (137.4 ± 12.4 pg/mL) and CAD + MS group: ≤ 50 years (342.7 ± 68.7 pg/mL), ≤ 70 years (448.4 ± 84.4 pg/mL), > 70 years (500.9 ± 155.9 pg/mL). Serum adiponectin levels in different groups were as follows; control group: ≤ 50 years (532.5 ± 23.6 µg/L), ≤ 70 years (491.9 ± 15.3 µg/L), > 70 years (478.7 ± 4.7 µg/L); CAD group: ≤ 50 years (379.4 ± 26.5 µg/L), ≤ 70 years (369.4 ± 21.9 µg/L), > 70 years (369.0 ± 19.2 µg/L); MS group: ≤ 50 years (454.3 ± 28.3 µg/L), ≤ 70 years (447.4 ± 23.7 µg/L), >70 years (454.4 ± 23.5 µg/L) and CAD + MS group: ≤ 50 years (245.2 ± 39.7 µg/L), ≤ 70 years (268.3 ± 22.4 µg/L), > 70 years (268.4 ± 16.6 µg/L). Serum LOX-1 and adiponectin levels of CAD and MS patients in different age groups between control and diseased groups were not statistically significant (P > 0.05). 

### 4.4. Serum LOX-1 Level in Different Groups

**Figure 1.**
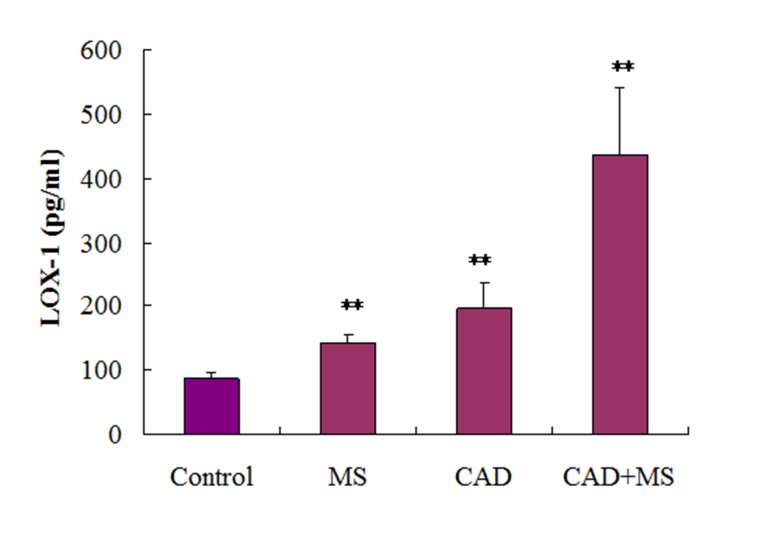
The highest LOX-1 level was found in CAD + MS group (435.9 ± 105.9 pg/mL), which was followed by CAD group (196.2 ± 40.4 pg/mL) and MS group (140.9 ± 15 pg/mL). In control group it was (87.1 ± 8.5 pg/mL). The result shows that the difference between control and diseased groups were statistically significant (P < 0.001).

Serum LOX-1 levels in different groups were shown in Figure 1. The highest LOX-1 level was found in CAD + MS group (435.9 ± 105.9 pg/mL), followed by CAD group (196.2 ± 40.4 pg/mL) and MS group (140.9 ± 15 pg/mL). It was 87.1 ± 8.5 pg/mL in control group. The differences between control and diseased groups were statistically significant (P < 0.001).

### 4.5. Serum Adiponectin Level in Different Groups

**Figure 2.**
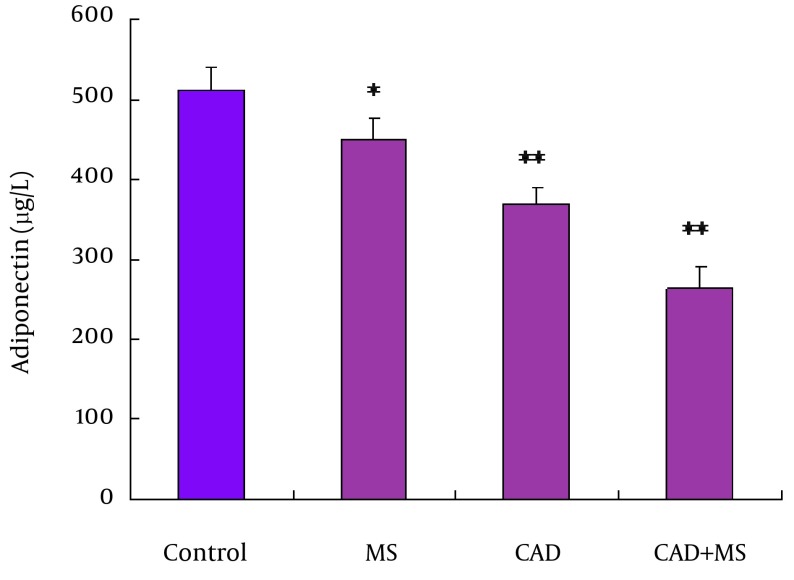
The lowest adiponectin level was found in CAD + MS (263.6 ± 26.6 µg/L) group, which was followed by CAD (369.9 ± 20.7 µg/L) group and MS (450.8 ± 25 µg/L) group. In control group it was (510.9 ± 29.3 µg/L). The result shows that the difference between control and diseased groups were statistically significant (P < 0.05, P < 0.001).

Serum adiponectin levels in different groups were shown in Figure 2. The lowest adiponectin level was found in CAD + MS (263.6 ± 26.6 µg/L) group, followed by CAD (369.9 ± 20.7 µg/L) group and MS (450.8 ± 25 µg/L) group. It was 510.9 ± 29.3 µg/L in control group. The differences between control and diseased groups were statistically significant (P < 0.05, P < 0.001).

### 4.6. Association Between LOX-1 and Adiponectin Levels in Different Groups

**Figure 3.**
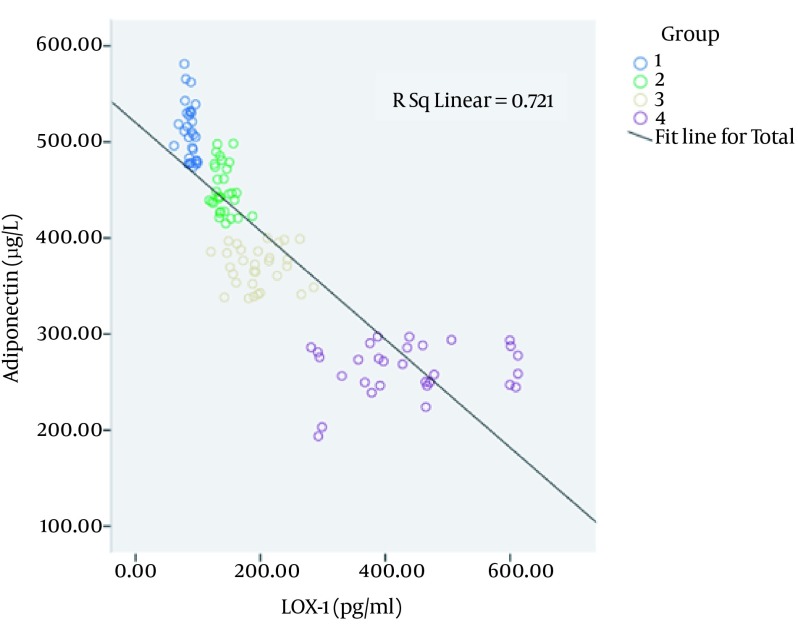
Plasma LOX-1 level was changed negatively and linearly (R2 = 0.721) correlated with adiponectin level in different groups.

Serum LOX-1 and adiponectin associations in different groups were shown in Figure 3. Serum LOX-1 level was changed negatively and linearly (R2 = 0.721) correlated with adiponectin level in different groups; the Pearson's correlation coefficient (r) was -0.849.

### 4.7. Association Between Adiponectin Level and Influencing Factors 

**Figure 4.**
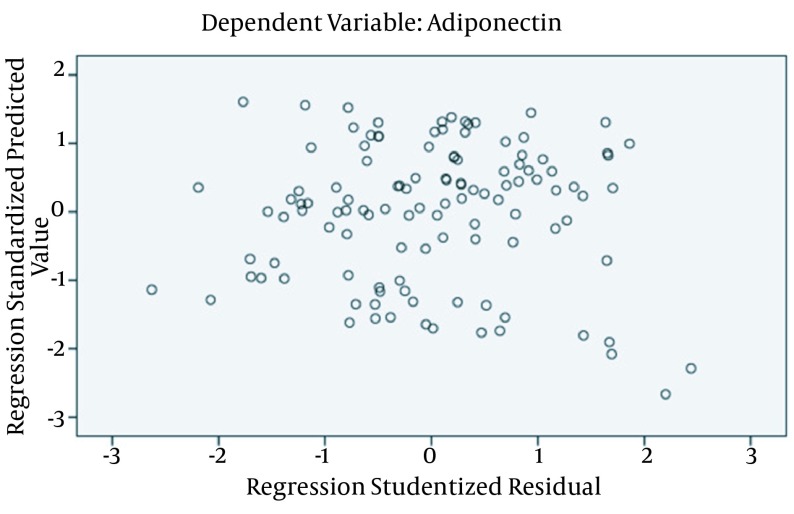
This study’s standardized residual plot showed that most points are located between ± 2 standard deviation and distributed uniformly around the 0 reference line, and it proved that these data basically met the conditions of multiple linear regression. But we should pay attention to the two suspicious points which located between -2 standard deviation and -3 standard deviation.

The association between adiponectin level and influencing factors was analyzed using multiple linear stepwise regressions method (α in = 0.05, α out = 0.10) considering adiponectin as the dependent variable, with age (≤ 50 years as 1, ≤ 70 years as 2, > 70 years as 3), gender (male as 1, female as 2), LOX-1, BMI, SBP, DBP, TG, TC, HDL-C, LDL-C, FBS, AST, ALT, BUN, Cr, UA, hs-CRP as independent variables. Results showed in Table 4, LOX-1, AST, BMI, TC, gender, BUN, DBP affected the Adiponectin level. Multiple correlation coefficient (R), coefficient of determination (R2), adjusted coefficient of determination, P values of this regression were 0.912, 0.832, 0.822, and < 0.001, respectively. We also judged the sample meet the conditions of multiple linear regressions through the standardized residual plot were shown in Figure 4.

**Table 1 .** General Clinical Information and Biochemical indicators of Patients and Control Groups ^a,b^

Parameters	Control	MS	CAD	CAD + MS	P Value
**Case, No.**	30	30	30	30	
**Age, y**	51 ± 11	53 ± 11	66 ± 9	61 ± 11	< 0.001
**Gender, %**					
Male	73	77	77	43	0.026
**BMI, kg/m** ^**2**^	21.1 ± 1.5	25.6 ± 1	23.7 ± 1.3	27.2 ± 1.3	< 0.001
**SBP, mmHg**	124 ± 8	136 ± 6	129 ± 10	142 ± 9	< 0.001
**DBP, mmHg**	72 ± 5	74 ± 7	77 ± 7	82 ± 7	< 0.001
**TG, mmol/L**	0.84 ± 0.3	2.3 ± 1.2	1.7 ± 1.6	2.1 ± 1.4	< 0.001
**TC, mmol/L**	4.2 ± 0.7	4.9 ± 1.1	4.1 ± 1.3	4.5 ± 1.1	0.015
**HDL-C, mmol/L**	1.7 ± 0.3	1.4 ± 0.3	1.4 ± 0.8	1.3 ± 0.4	0.001
**LDL-C, mmol/L**	1.9 ± 0.6	2.4 ± 0.9	2.3 ± 1.0	2.3 ± 1.0	0.156
**FBS, mmol/L**	4.5 ± 0.3	5.9 ± 0.6	5.0 ± 0.7	6.2 ± 0.7	< 0.001
**AST, U/L**	11.2 ± 3.6	18.3 ± 4.3	21.2 ± 5.4	23.5 ± 4.8	< 0.001
**ALT, U/L**	14.5 ± 4.5	19.0 ± 4.0	20.9 ± 5.6	21.2 ± 4	< 0.001
**BUN, mmol/L**	3.9 ± 0.6	4.8 ± 1.4	5.5 ± 0.9	5.3 ± 1	< 0.001
**Cr, mg/L**	71.0 ± 9.8	83.5 ± 10.6	85.8 ± 14.1	89.3 ± 16	< 0.001
**UA, mmol/L**	251.9 ± 42	307.0 ± 60.0	285.7 ± 53.5	314.7 ± 55.1	< 0.001
**hs-CRP, mg/L**	0.7 ± 0.8	1.7 ± 2.9	7.2 ± 9.8	12.0 ± 8.5	< 0.001

^a^ Results were mean ± SD. Values in a same row with different superscripts were significantly different (P < 0.05).

^b^ Abbreviations: ALT; alanine transaminase, AST; aspartate aminotransferase, BMI; body mass index, BUN; blood urine nitrogen, CAD; coronary artery disease, Cr; creatinine, DBP; Diastolic blood pressure, FBS; fasting blood sugar, LDL; low density lipoprotein, HDL; high density lipoprotein, MS; metabolic syndrome, SBP; systolic blood pressure, TG; triglyceride, TC; total cholesterol, UA; uric acid.

**Table 2.** LOX-1 and Adiponectin Levels of Male and Female in Different Groups ^a,b^

	Control	MS	CAD	CAD + MS
**LOX-1 (pg/mL)**				
Male	86.6 ± 8.2	137.2 ± 11.5	195.0 ± 41.5	464.4 ± 100.8
Female	88.7 ± 9.9	149.7 ± 18.8	200.0 ± 39.4	414.3 ± 107.5
P-value	0.557	0.033	0.779	0.205
**Adiponectin (µg/L)**				
Male	515.1 ± 30.0	456.9 ± 23.8	370.5 ± 20.9	264.8 ± 20
Female	499.1 ± 25.4	436.7 ± 23.1	368.0 ± 21.4	262.8 ± 31.4
P-value	0.191	0.041	0.786	0.839

^a^ Values were mean ± SD.

^b^ CAD; coronary artery disease, MS; metabolic syndrome.

**Table 3.** LOX-1 and Adiponectin Levels in Different Groups With Different Ages ^a,b^

	Control	MS	CAD	CAD + MS
**LOX-1, pg/mL**				
≤ 50 y	85.5 ± 5.5	139.4 ± 11.1	232.4 ± 8.1	342.7 ± 68.7
≤ 70 y	87.8 ± 11.5	142.9 ± 18.3	178.6 ± 28.6	448.4 ± 84.4
> 70 y	92.5 ± 6.8	137.4 ± 12.4	220.5 ± 46.3	500.9 ± 155.9
P-value	0.420	0.766	0.018	0.039
**Adiponectin, µg/L**				
≤ 50 y	532.5 ± 23.6	454.3 ± 28.3	379.4 ± 26.5	245.2 ± 39.7
≤ 70 y	491.9 ± 15.3	447.4 ± 23.7	369.4 ± 21.9	268.3 ± 22.4
> 70 y	478.7 ± 4.7	454.4 ± 23.5	369.0 ± 19.2	268.4 ± 16.6
P-value	< 0.001	0.763	0.809	0.165

^a^ Values were mean ± SD. The difference of LOX-1 between different age groups in CAD + MS and CAD groups was assessed using Kruskal-Wallis H and Student-Newman-Keuls post hoc tests.

^b^ CAD; coronary artery disease, MS; metabolic syndrome.

**Table 4.** Multiple Linear Regression Regarding Adiponectin Level and Influencing Factors ^a^

	*b*	*s*_*b*_	*b’*	*t*	P Value
**Constant**	802.645	56.451		14.218	< 0.001
**LOX-1, pg/mL**	-0.352	0.038	-0.531	-9.355	< 0.001
**AST, U/L**	-3.043	0.766	-0.204	-3.792	< 0.001
**BMI, kg/m** ^**2**^	-6.214	2.066	-0.166	-3.008	0.003
**TC, mmol/L**	12.752	3.508	0.147	3.636	< 0.001
**Gender**	-26.878	8.280	-0.133	-3.246	0.002
**BUN, mmol/L**	-9.355	3.649	-0.114	-2.564	0.012
**DBP, mmHg**	-1.226	0.533	-0.100	-2.301	0.023

^a^ Abbreviations: AST; aspartate aminotransferase, BMI; body mass index, BUN; blood urine nitrogen, DBP; Diastolic blood pressure, TC; total cholesterol.

## 5. Discussion

CAD is the most common type of heart disease, which is usually caused by atherosclerosis. Increased plasma level of low-density lipoprotein is a well-known risk factor for endothelial dysfunction and atherosclerosis. The pro-atherosclerotic potential of low density lipoprotein may even increase after oxidative modification to ox-LDL, whose uptake by macrophage scavenger receptors is thought to be a key process in the formation of foam cells, as the hallmark of atherosclerotic lesions (17). In human atherosclerotic lesions, LOX-1 is expressed prominently by intimal smooth muscle cells and lipid-laden macrophages in advanced plaques. Furthermore, LOX-1 plays an important role in oxidized-low density lipoprotein induced apoptosis of vascular smooth muscle cells and production of matrix metalloproteinases, which might be directly associated with plaque rupture. LOX-1 is also expressed on the surface of activated platelets, which may also be involved in thrombus formation after plaque rupture (18). Although LOX-1 is expressed at very low levels in healthy endothelium, several lines of evidence support a role of LOX-1 in the pathogenesis of atherosclerosis (19). Meanwhile, LOX-1 expression is increased in hypertension, diabetes mellitus, hyperlipidemia, chronic renal failure, acute coronary syndromes and the highest expression of LOX-1 is found in atherosclerotic lesions (20). Several research groups have analyzed the association between LOX-1 gene polymorphisms and CAD and a higher risk of acute myocardial infarction (21). A community-based cohort study, measured apolipoprotein B and soluble LOX-1 using ELISA with recombinant LOX-1 and monoclonal anti-apolipoprotein B antibody and with two monoclonal antibodies against LOX-1, respectively; higher Lectin-like oxidized-low density lipoprotein index [Lectin-like oxidized-low density lipoprotein index apolipoprotein B × soluble LOX-1] values were associated with increased risk of coronary heart disease (22). Generally LOX-1 level has a positive association with vascular inflammation. In the present study, the highest LOX-1 level was found in CAD + MS group, followed by CAD group and MS group. It was the lowest in control group. Our finding of gradually increasing serum LOX-1 concentration with increasing severity of CAD was consistent with some other studies (5, 21-23). Most studies investigated the impact of LOX-1 on the incidence of CAD, but in addition to CAD, we tried to find out the role of LOX-1 in CAD plus MS and only MS patients. Our finding suggests CAD plus MS group as the highest risk group for acute myocardial infarction; the severity increased more than two folds compared to CAD group and more than four folds compared to control group. Whereas, patients in MS group had LOX-1 expression more than one fold as compared to control group.

Adiponectin is one of the important active cytokines secreted from adipose tissue, which regulates glucose and lipid homeostasis, energy metabolism, and poses anti-inflammatory activity (24). Dysregulation of adiponectin has been implicated in metabolic X syndrome, atherosclerosis, obesity, hypertension and CAD (25). Atherosclerotic endothelial dysfunction, particularly in the early disease stages, is primarily due to dysregulation of endothelial nitric oxide synthase enzymatic activity and inactivation of nitric oxide through oxidative stress (26). Reduced bioavailability of nitric oxide is involved in the initiation, progression and complications of atherosclerosis. Adiponectin induces endothelial nitric oxide synthase activation and nitric oxide production in endothelial cells. Adiponectin reduces reactive oxygen species production as well as improving endothelial function in aortas of ApoE KO mice (27). Adiponectin can potentially inhibit all the molecular pathways of atherosclerosis, including monocyte adhesion to endothelial cells by adhesion molecules, ox-LDL uptake of macrophages through scavenger receptors and proliferation of migrated smooth muscle cells by the action of platelet-derived growth factors and heparin-binding epidermal growth factor. Therefore, it seems that adiponectin is predominantly beneficial and high levels of circulating adiponectin confer vascular protection (28). Adiponectin has beneficial roles for the vascular system and it has been proven that decreased plasma levels of adiponectin are associated with coronary atherosclerosis (29). Low serum adiponectin levels are significantly correlated with endothelial dysfunction. Hypoadiponectinemia correlates significantly and independently with CAD. Serum concentrations of adiponectin in patients with acute coronary syndromes are significantly lower than those with stable angina and the control group. Individuals with adiponectin concentrations in the highest quintile compared with the lowest quintile have a decreased risk of myocardial infarction (30). In addition, adiponectin was associated with a decreased risk of coronary heart disease events in the same cohort men with diabetes. However, adiponectin has a negative association with vascular inflammation (31). In this study, we found that adiponectin level is significantly different between diseased and control group. The lowest adiponectin level was found in CAD + MS group, which was followed by CAD group and MS group. It was the highest in control group. Our results indicated that decreasing serum adiponectin level is associated with increasing severity of CAD. According to our findings, CAD plus MS group was the most vulnerable group followed by CAD group and MS group as compared to control group. Similar findings were found in many other studies (10, 13, 26, 29, 30, 32).

According to Arita et al. women had about 40% higher circulating levels of adiponectin than men, but in our study adiponectin levels of male and female between control and diseased groups were not statistically different (P = 0.839). We determined the association between LOX-1 and adiponectin in CAD and CAD + MS patients. Our study showed that plasma LOX-1 level was changed negatively and linearly (R2 = 0.721) correlated with adiponectin level in different groups. Our results also showed that LOX-1, AST, BMI, TC, gender, BUN, DBP affected Adiponectin level. Multiple correlation coefficient (R), coefficient of determination (R2), adjusted coefficient of determination and P value of this regression were 0.912, 0.832, 0.822 and < 0.001, respectively.

Furthermore, low adiponectin levels were associated with MS. MS has received increasing attention because it represents an important risk for cardiovascular diseases (32). MS is a compilation of associated risk factors, increases the risk of CAD, which can progress to artery occlusion (33). Our finding also suggests that low level of adiponectin and high level of LOX-1 were related to MS. Our findings are in agreement with some others (33, 34). There are very few studies regarding the evaluation of these two parameters. According to our knowledge, we determined and evaluated this inverse association in patients with CAD and CAD + MS for the first time.

In conclusion, we found that serum levels of LOX-1 are increased in CAD, but this increase is more obvious in patients with MS. Serum levels of adiponectin are decreased in CAD and the decrease is more remarkable in patients with MS. Our findings showed for the first time that using combination of these two important serum parameters may be an effective method to evaluate the severity of cardiovascular disease accompanied with metabolic disease such as CAD plus MS. Patients with CAD and MS seem to have a higher risk than those with only CAD because of both lipid and glucose metabolism abnormalities. The result may not be novel but of clinical importance. More patients should be enrolled and more cardiovascular events such as cardiovascular mortalities and cardiovascular hospitalization rates should be assessed in future studies to evaluate the effect of LOX-1 and adiponectin.
